# Teachers in Conference

**Published:** 1921-01-15

**Authors:** 


**THE PUBLIC WELL-BEING—(*continued*).**

### Teachers in Conference.

### A CANDID SPEAKER AND A PROTEST.

**By A Social Worker.**

UNIVERSITY COLLEGE, London, has been since December 29 and continued to be until January 8 a humming hive of teachers, educationists, and administrators of education. Forty-six educational associations were holding conference, admitting each other's members to all, but their business meetings, and in some cases conferring jointly when papers were read by eminent specialists. Some of these papers were, as usual, very interesting, and one leading note this year—such gatherings have always some distinctive notes—was the enormously increased interest taken by teachers as well as by educationists in the physical and mental health of the child. Indeed, so far, the most novel ideas prescribed are, first, Dr. Tredgold's contention, in addressing the Eugenics Education Society, that paucity of brain-power, apart from actual feeble-mindedness, is a quality that must be reckoned with in a number of children. Much, therefore, that is said and done to give equal opportunity to every child must of necessity be futile.

Secondly, a morning devoted to Parents' Committees by the Civic Education League put the parents, so long the object of reproach, sarcasm, or neglect from teachers, in a new and better light than that of mere providers of raw material for schools. Both teacher and parents will gain by every effort made to harness them together to the educational coach, while to some children at least the relief of not being pulled in two directions should be a primary good. Incidentally, the school nurse and doctor should be helped in their work.

PSYCHO-ANALYSIS.

A joint conference which took Psycho-Analysis for its subject drew so large a crowd that many were turned away. Dr. H. Crichton Miller impressed upon the audience that the child's great need is spiritual freedom for harmonious development. Organised researches into the sub-consciousness of the normal child are not needed, but to the teacher analysis by self or another would be wholesome if it gave clearer vision. He deprecated the craze for mutual research that has broken out here and there among young people in colleges. Reading in moderation he recommended, mentioning especially Dr. Bernard Hart's "Psychology of the Insane," and Dr. Constance Long's volume of essays, collected under the title of the "Psychology of Sanity."

Mr. George Green, of the Hornsey County School, spoke of a study made of the dreams of children preparing for an examination. Of thirty-two children twenty-two dreamed, one of a high place, eleven with dread. A successful child gains elation; work is then full of hope and pleasure. An unsuccessful one may be hindered by undiscovered myopia. Another may have an unlucky habit of working with pains, then blotting a paper by clumsi-

**THE PUBLIC WELL-BEING**—(*continued*).

ness and being set down as careless. Both are discouraged; the discouraged child becomes fidgety, noisy, difficult. Such children dread tests, they also suffer from mind wandering into day-dreams of high fortune and distraction. A teacher whose mind is awakened to psycho-analysis should approach school problems from a new and helpful standpoint.

A considerable audience also gathered to hear Miss Norah March speak on sex biology with especial reference to care of the child and adolescent. The lecture, however, consisted merely of the biological facts of sex and reproduction, stated absolutely without reserve to the mixed audience, some of whom protested by leaving the room. The question is undoubtedly a very difficult one; on the one hand those thinkers whom Miss March represents seek to abolish the evils that arise from youthful curiosity by leaving nothing to be curious about. On the other hand it is at least questionable whether modesty and reticence on certain subjects are qualities which should be entirely discouraged.

The treatment of the young criminal was the subject of an admirable address to the Associations of Head Mistresses and Assistant Mistresses by Miss Margery Fry, of the Penal Reform League. Her contention that medical examination should be applied to discover any physical contributory causes to a dangerous frame of mind, especially in the case of foolish crime often repeated, was strongly approved, and resolutions were framed to be sent to the Home Office, the Board of Education, and the Ministry of Labour, urging the establishment of a national system of probation on unsectarian lines, the cessation of confinement of young people under twenty-one in jails for older criminals, and establishment of public control for all reformatories and industrial schools.

